# Successful endoscopic ultrasound-guided nasocavitary catheter drainage of abscess caused by delayed perforation after gastric endoscopic submucosal dissection

**DOI:** 10.1016/j.vgie.2021.12.011

**Published:** 2022-01-26

**Authors:** Shunya Takayanagi, Maiko Takita, Ken Ishii, Yuji Fujita, Ken Ohata

**Affiliations:** 1 Department of Gastroenterology, NTT Medical Center Tokyo, Tokyo, Japan; 2 Sakura Naishikyo Clinic Shinagawa, Tokyo, Japan; 3 Department of Gastroenterology, National Hospital Organization Yokohama Medical Center, Kanagawa, Japan

**Keywords:** ESD, endoscopic submucosal dissection, POD, postoperative day

## Abstract

Video 1 Successful EUS-guided nasocavitary catheter drainage of abscess caused by delayed perforation after gastric endoscopic submucosal dissection.

Successful EUS-guided nasocavitary catheter drainage of abscess caused by delayed perforation after gastric endoscopic submucosal dissection.

Delayed perforation after gastric endoscopic submucosal dissection (ESD) is a rare but serious adverse event often requiring emergency surgery.^1^^,^^2^ Here, we report the use of EUS-guided nasocavitary catheter drainage to treat an intra-abdominal abscess caused by delayed perforation after gastric ESD.

A 78-year-old man with early gastric cancer in the lesser curvature of the stomach underwent ESD with no intraprocedural adverse events (Fig. 1A and B). However, on postoperative day (POD) 5, he reported fever and abdominal discomfort. EGD showed a 5-mm perforation in the mucosal defect (Fig. 2A), and abdominal CT revealed a fluid collection around the lesser curvature of the stomach (Fig. 2B). The patient was diagnosed with intra-abdominal abscess caused by a delayed perforation. Because his symptoms were mild, he was managed conservatively with antibiotics, a proton pump inhibitor, parenteral nutrition, and attachment of a polyglycolic acid sheet to the perforated ulcer (Fig. 3).

**Figure 1:**
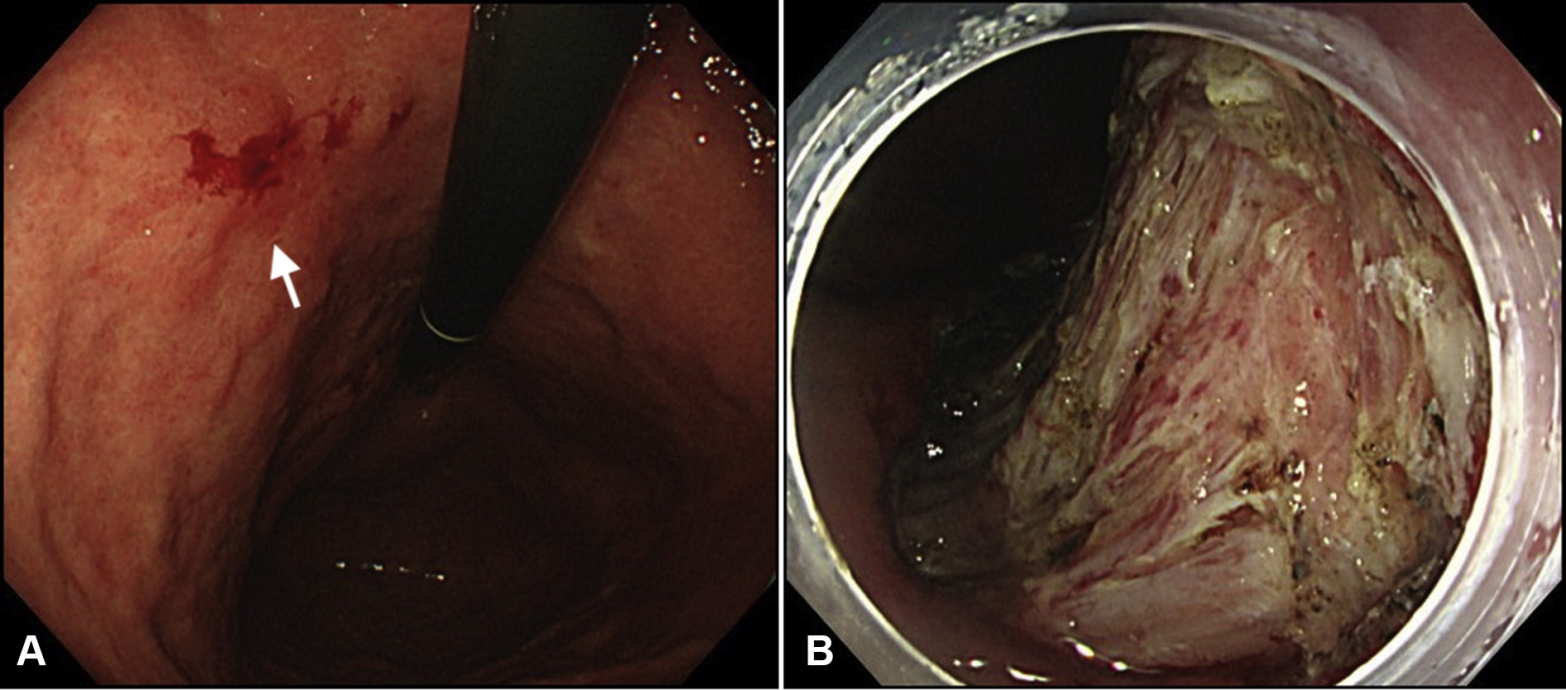
Endoscopic findings. **A,** The lesion (*white arrow*) was located in the lesser curvature of the stomach. **B,** Endoscopic submucosal dissection was performed without intraoperative perforation.

**Figure 2:**
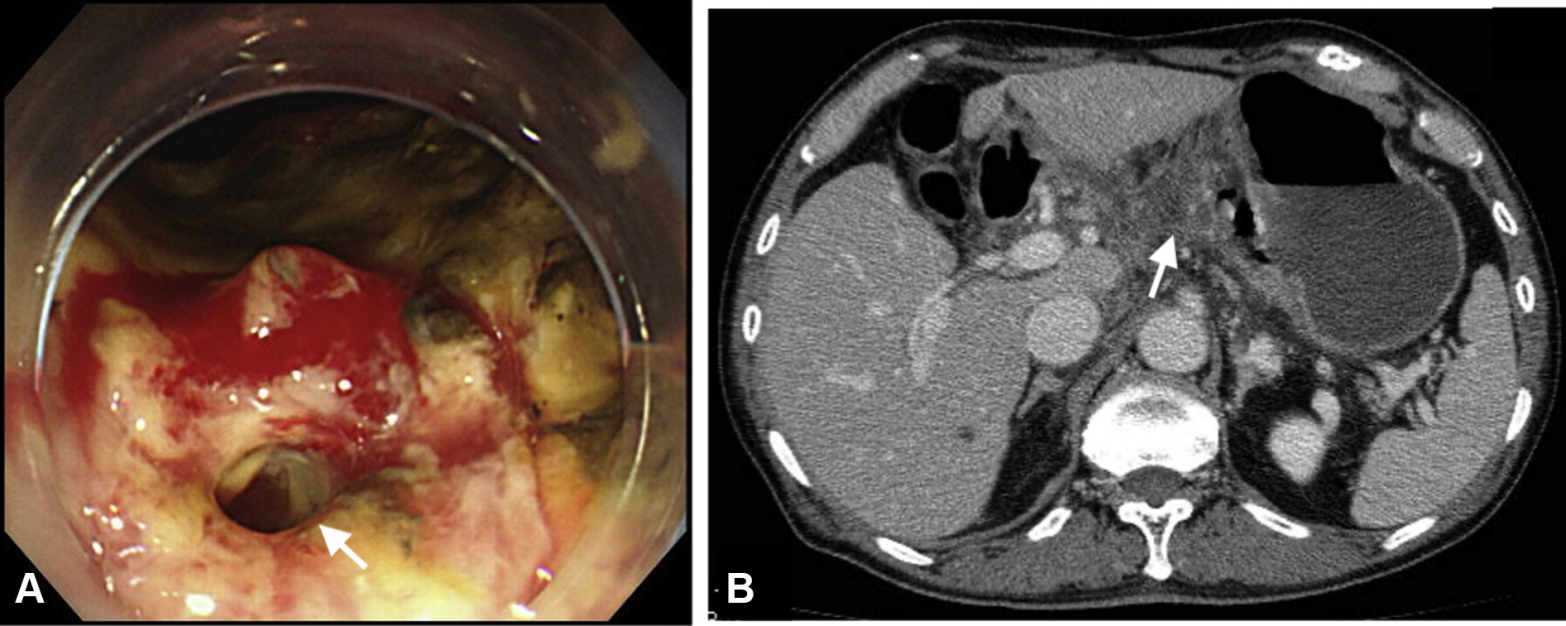
Delayed perforation occurred on postoperative day 5. **A,** A fistula (*white arrow*) in the post–endoscopic submucosal dissection ulcer was observed. **B,** Abdominal CT revealed fluid collection (*white arrow*) around the lesser curvature of the stomach.

**Figure 3:**
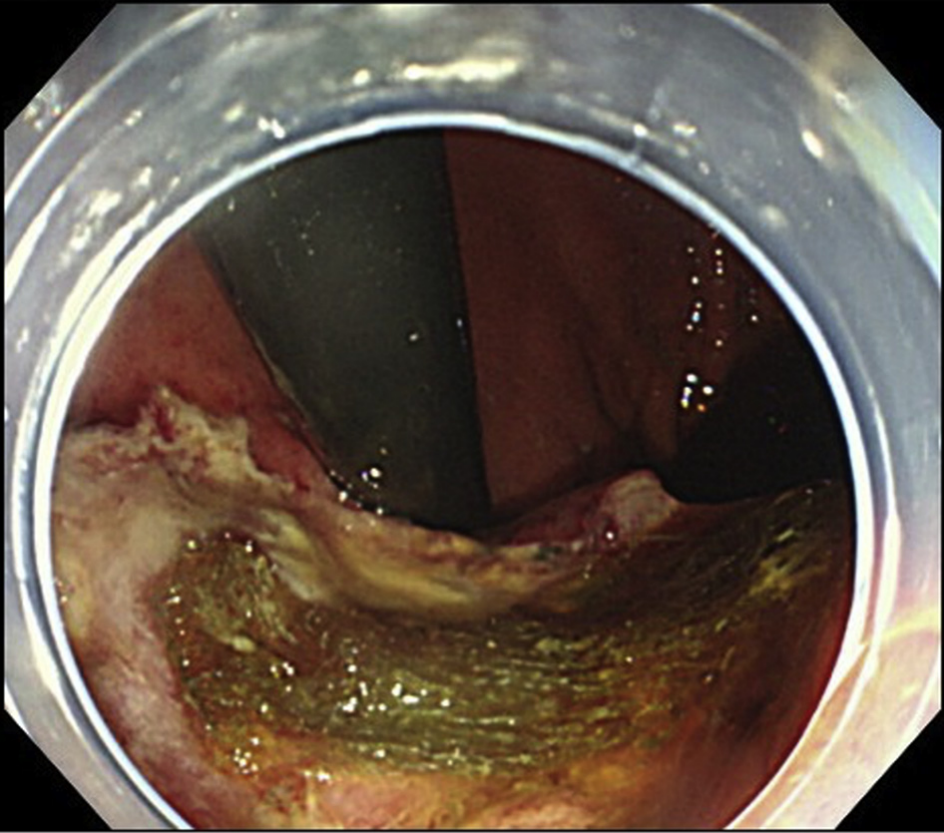
The perforated ulcer was closed with a polyglycolic acid sheet.

A follow-up EGD on POD12 showed closure of the fistula; however, the patient had a slight persistent fever, and a follow-up CT showed continued presence of the abscess (Fig. 4).

**Figure 4:**
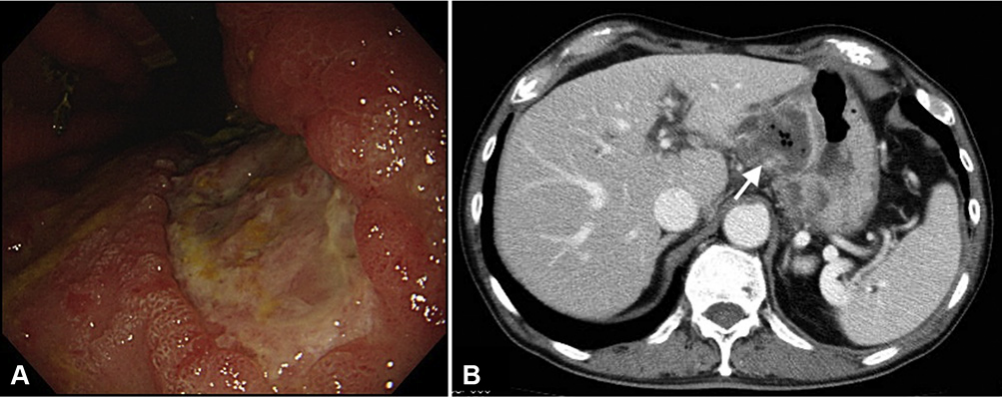
Follow-up endoscopic and CT findings on postoperative day 12. **A,** The perforation was closed. **B,** The intra-abdominal abscess (*white arrow*) is still present.

Because closure of the fistula could prevent drainage of the abscess and to promote the formation of a pyogenic membrane, further conservative treatment was not considered effective. Although surgical drainage was considered, EUS-guided drainage was performed because it was a less invasive treatment. The 50-mm abscess adjacent to the gastric wall was visualized using EUS. A 19-gauge needle (EZ Shot 3 Plus; Olympus Medical, Tokyo, Japan) was inserted into the abscess under continuous EUS and fluoroscopic guidance, and a 0.025-inch guidewire (Visiglide2; Olympus Medical) was inserted through the needle, followed by the insertion of a 6F nasobiliary drainage tube (SilkyPass; Boston Scientific, Marlborough, Mass, USA) into the cavity via surrounding normal mucosa (Fig. 5; Video 1, available online at www.giejournal.org). The patient’s symptoms disappeared within 2 days. A follow-up CT on POD34 revealed complete resolution of the abscess (Fig. 6), and the tube was removed. The patient was discharged on POD38 without further adverse events.

**Figure 5:**
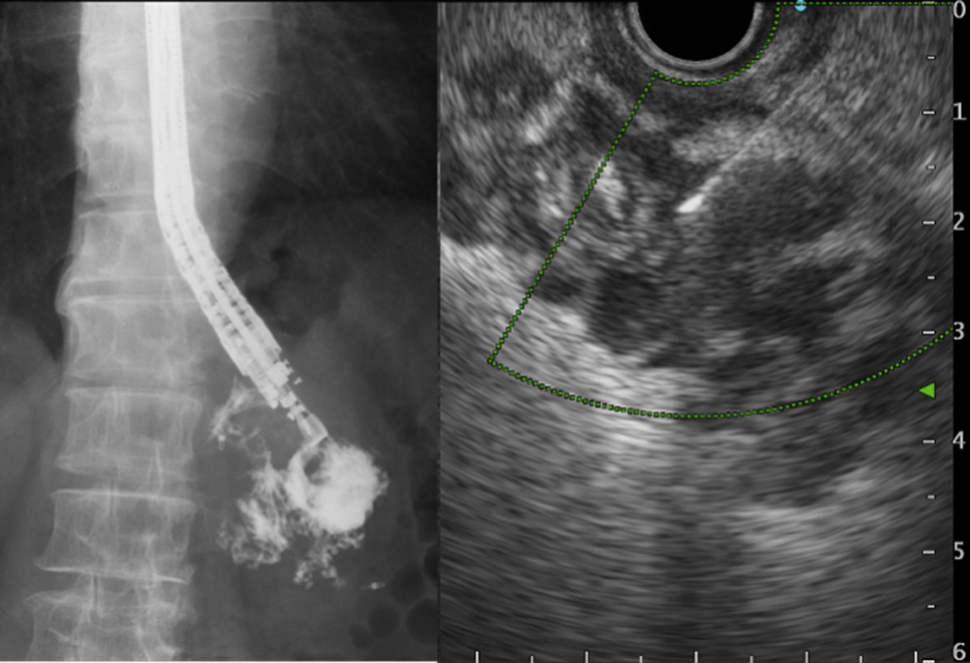
EUS-guided nasocavitary catheter drainage of the abscess was performed.

**Figure 6:**
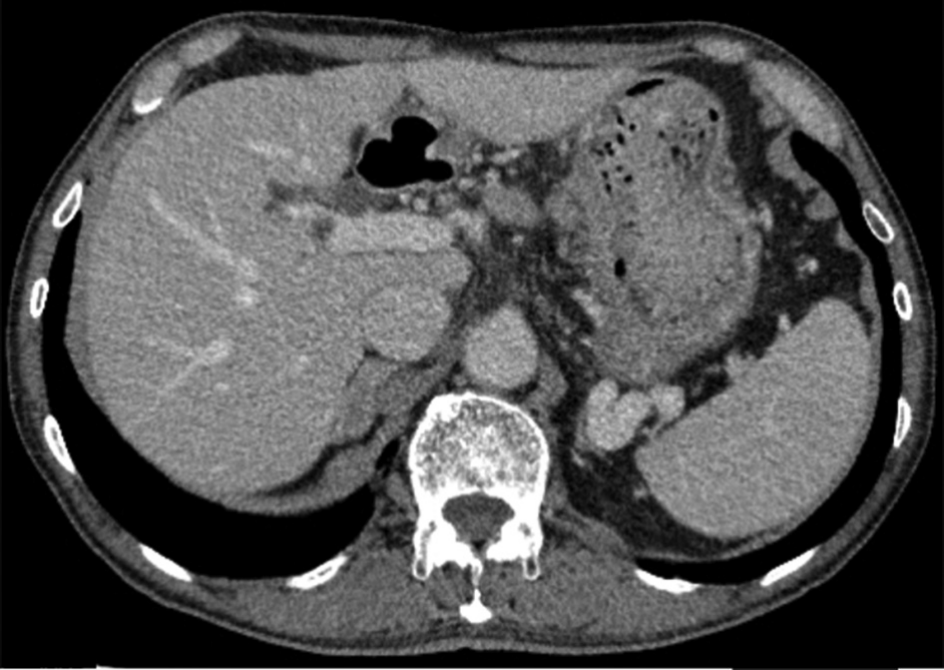
Posttreatment enhanced abdominal CT on postoperative day 34 showed that the abscess had completely resolved.

Generally, antibiotics poorly penetrate an established abscess; hence, surgery and percutaneous drainage are often required.^3^ Percutaneous drainage is often used as a first-line treatment for intra-abdominal abscesses^4^ and is a less invasive treatment than surgical drainage.^5^ Because intra-abdominal abscesses caused by perforation after gastric ESD are thought to be located adjacent to the gastric wall, especially when the fistula is closed and spontaneous drainage is not expected, drainage via the gastric wall is more effective and direct than percutaneous drainage. However, because intra-abdominal abscesses can sometimes lead to a fatal consequence, we must keep in mind that immediate surgical intervention is required if infection is not controlled after EUS-guided drainage.

EUS-guided nasocavitary catheter drainage can be performed as an alternative treatment for delayed perforation after gastric ESD.

## Disclosure

*All authors disclosed no financial relationships.*

## Supplementary data

Video 1 Successful EUS-guided nasocavitary catheter drainage of abscess caused by delayed perforation after gastric endoscopic submucosal dissection.
